# Comparison of Neck Injury Criteria Values Across Human Body Models of Varying Complexity

**DOI:** 10.3389/fbioe.2020.00985

**Published:** 2020-08-18

**Authors:** Dale Johnson, Bharath Koya, F. Scott Gayzik

**Affiliations:** ^1^Center for Injury Biomechanics, Wake Forest University, Winston-Salem, NC, United States; ^2^Department of Biomedical Engineering, Wake Forest University, Winston-Salem, NC, United States

**Keywords:** human body model, neck injury, Biomechanics, injury criteria, GHBMC

## Abstract

Due to the severity and frequency of cervical spine injuries, the neck injury criterion (Nij) was developed to provide a quantitative relationship between forces and moments of the upper neck with accompanied injury risk. The present study was undertaken to evaluate differences in calculated Nij for the Global Human Body Model Consortium’s detailed and simplified average 50th percentile male models. The simplified model is a computationally light version of the more detailed model and therefore it is of interest to achieve similar Nij values between the two models. These models were compared in 15 match paired conditions of rigid head impact and a mixture of seven full body rigid hub and sled pulses, for 44 total simulations. Collectively, Nij values of the simplified model were found to exhibit a second-degree polynomial fit, allowing for a conversion to the prediction of the detailed model. Correlates were also derived for impact and inertial loading cases individually, for which the latter may be the subject of future work. The differences in Nij may be attributed to a variety of modeling approach differences related to neck muscles (attachment location and morphometric implementation), localization of head mass within the M50-OS, head geometry, and intervertebral joint space properties. With a primary focus on configurations in the anterior-posterior direction, there is a potential limitation in extensibility to lateral loading cases. In response to the relatively low Nij values exhibited, future work should evaluate the appropriateness of the established critical intercepts of Nij for computational human body models.

## Introduction

Between 2005 and 2013, nearly half a million cases of cervical spine fracture were seen nationally, along with a trend of increased incidence from 4.1 to 5.4% (Passias et al., 2018). In the aforementioned study, a majority of these injuries consisted of C2 (32%) and C7 (20.9%) closed fractures, were fatal in 15% of cases, and were most commonly caused by motor vehicle crashes. Similar distributions of cervical spine fracture based upon vertebral level have been reported (Goldberg et al., 2001). In an analysis of over 50,000 trauma cases, most spinal cord injuries occurred at the cervical spine level and 93% resulted in Abbreviated Injury Scale (AIS) 3+ outcomes (Stephan et al., 2015). An AIS score of 1–6 corresponds to injuries of a body region that are minor, moderate, serious, severe, critical, and maximal (untreatable) (“Association for the Advancement of Automotive Medicine, 2018). Therefore, the instance of an isolated, closed fracture of the cervical spine could present as low as an AIS 2 or 3 injury. However, at the insult of the spinal cord or other prominent anatomy, AIS scores quickly rise. At the upper neck region in particular, a majority of fractures occur due to low facial impacts that induce large extension-tension forces (Passias et al., 2018).

To evaluate the efficacy of modern safety systems in motor vehicles and other applications, the Neck Injury Criteria (Nij) was proposed and then revised in 2000 (Eppinger et al., 2000). The calculation of Nij is shown in Eq. 1 below as a combination of axial force (*F*_*z*_) and sagittal moment (*M*_*y*_) normalized by an axial force critical intercept (*F*_*zc*_) and sagittal moment intercept (*M*_*yc*_). The critical intercepts for axial compression (Mertz’s et al., 1978) and tension (Nyquist et al., 1980) were determined from loading configurations of the Hybrid III 50 percentile male anthropomorphic test device (ATD). In compression, the simplified neck of the Hybrid III has been shown to display a stiffer response compared to volunteer and cadaver cervical spines (Wismans et al., 1987; Yoganandan et al., 1989; Nightingale et al., 1991; Svensson and Lövsund, 1992). However, a statistical analysis of multiple cadaveric cervical spine compression studies identified a similar critical intercept (Pintar et al., 1998) to that which was previously established by Mertz’s et al. (1978) interrogation of the Hybrid III. Flexion-extension tolerances were determined by volunteer and cadaver sled tests (Mertz and Patrick, 1971), with volunteer data provided up to a pain threshold. Critical intercepts for extension and flexion were determined by experimental sled tests with volunteers and cadaver subjects. For extension, ligamentous injury of a small stature cadaver was scaled up to a 50th percentile male. In flexion, no injury was exhibited up to a maximum measurement of 190 Nm.

The current intercepts of compression/tension and flexion/extension for an in position 50th percentile male occupant are 6160/6806 N and 310/135 Nm, as well as peak compression/tension forces of 4000 and 4170 N. While cadavers are used in some capacity to establish injury metrics for the neck, they lack the ability to be directly instrumented for loading in the upper neck region. Often, cadaveric studies require additional fixation devices and simplified loading conditions (Sances et al., 1982; Panjabi et al., 1991; Yoganandan et al., 1991). A final correlation between Nij and various AIS injury risks was determined based on porcine data (Prasad and Daniel, 1984; Mertz et al., 1987). From this correlate, an Nij value of 1 coincides with a 22% risk of an AIS 3+ injury.

$$N\,i\,j=\frac{F_{z}}{F_{z\,c}}+\frac{M_{y}}{M_{y\,c}};$$

where

(1) $$F_{z}=\text{upper}\,\text{neck}\,\text{force},M_{y}=\text{Total}\,\text{Moment}$$

The investigation of neck injury for applications of occupant protection stands to benefit greatly from the use of FE human body models. This is because human models do not need to rely on correlative measures to determine neck forces and moments based on acceleration and assumed head mass or potential interactions of instrumentation devices. It can be determined directly through model interrogation. Validation into the biofidelity of human models must precede any predictive capabilities that may be leveraged. The specific scope of this work is related to the Global Human Body Model Consortium (GHBMC) detailed (M50-O) and simplified (M50-OS) average 50th percentile male occupant models as they compare to one another in terms of neck anatomical architecture, Nij, and kinematics of the head and neck. The neck bony geometry of each model was determined from the same source, an average male volunteer who matched target anthropometry (Gayzik et al., 2011). Outside of the bony geometry, the outer surface of the skin for the M50-O and M50-OS is equivalent. Remaining anatomical features of each model have been developed independently of one another and will be further detailed below. Regarding the detailed model, development of the neck includes bones, ligaments, active and passive muscle, flesh, and intervertebral discs. The three dimensional mesh of the intervertebral discs in the M50-O is comprised of material laws for both the annulus fibrosus and nucleus pulposus. The M50-O neck has been validated in linear impact for passive and active musculature (Bruneau and Cronin, 2019), deviation from nominal occupant position postures at the whole body (Gayzik et al., 2018) and tissue level (Shateri and Cronin, 2015), and exhibited close agreement to PMHS data in a study of a frontal, restrained occupant (Schap et al., 2019).

The simplified model neck bony geometry is identical to that of the detailed model but additional anatomy was modeled based on previously established methodology (Dibb, 2011, pg. 36–53). The specific means by which the M50-OS employs the previous method is through representation of muscle as a single stranded 1D element that extends from origin to insertion without intermediate points of attachment. A key distinction between the M50-O and M50-OS neck models is the substitution of deformable vertebral bodies in the M50-O, for rigid bodies in the M50-OS. Due to modeling approach differences in the material of the cervical vertebrae, the M50-OS requires contribution of 6 degree-of-freedom, zero length beams, in addition to discrete elements (spring and dampers) for X, Y, and Z directions, between vertebrae (representing intervertebral discs) as a surrogate from the otherwise absent contribution of the vertebrae.

A point that warrants further discussion is related to the simplification of the muscle tissue from detailed to simplified models. While the M50-O uses a combination of 3D elements and 1D beams, the M50-OS relies on 1D beams between origin and insertion points. Pertaining to the 1D beams, origin and/or insertion points which occur on bony aspects of the models are identical between the M50-O and M50-OS. However, points which attach outside of the skeletal structure may present with subtle differences between the models. Aside from origin and insertion, the 1D beams of the M50-O contain intermediate nodes that are constrained to the associated 3D mesh and also the vertebral bodies by 1D discrete springs. The M50-OS muscles are single stranded and therefore do not contain intermediate points, as there is no 3D mesh surrounding the osteoligamentous spine to adhere them to in an equivalent manner. The physiological cross sectional area (PCSA) of the M50-O and M50-OS are similar. For example, the PCSA’s of the M50-O and M50-OS for the sternocleidomastoid (483.1 and 492 mm), anterior scalene (187.3 and 188 mm), and inferior oblique capitis (195.1 and 195 mm) are provided, respectively. Despite these differences, both models have been shown to have a fair correlation, based on an objective evaluation analysis, ISO TS 18571 (Barbat et al., 2013), with volunteer data in terms of head linear and rotational acceleration, as well as T1 linear acceleration in a restrained frontal impact sled test condition (Decker et al., 2017). In the work of Decker et al. (2017), the ISO score difference in frontal kinematic response of the M50-O and M50-OS was marginal for head rotational acceleration and <0.1 for linear head and T1 acceleration. The –OS model performed well in omnidirectional impact loading versus volunteer data for the following metrics: head resultant acceleration, sternum X acceleration, and belt forces of the left and right lap and shoulder belts, receiving a fair rating by CORA analysis (Gaewsky et al., 2019). CORA is another objective evaluation method which returns a cross correlation score based on size, shape, phase, and corridor (Gehre et al., 2009). A recent study, part of which utilized 4 impact conditions [both sagittal (*n* = 3) and lateral (*n* = 1)] noted a lack of direct comparison between the neck moments of the M50-O and M50-OS based on magnitude and temporal domains, especially when extension and lateral bending of the neck were induced (Jones, 2019).

The simplified model runs roughly 30–40 times faster than the detailed model on similar hardware (Schwartz et al., 2015) and is ideal for large scale parametric studies. Due to its greater anatomical biofidelity, the detailed model is viewed as the standard compared to its simplified counterpart. There remains a gap in how divergent Nij values calculated via the simplified model are from the detailed model. Therefore, while each model has been validated for head kinematics, there has been relatively little study on the common injury metric, Nij, as it compares between the two. This study aims to provide preliminary data on the comparative capabilities for Nij of the aforementioned models.

## Methods

**FIGURE 1:**
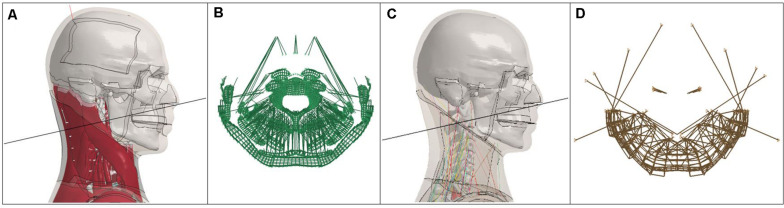
**(A,B)** M50-O (left half) and **(C,D)** M50-OS (right half) C2 section plane and cross section.

The GHBMC M50-O and M50-OS, versions 5.0 and 2.1, respectively, were used as the basis for this work. Each model’s version was held consistent, unless otherwise noted, and positioned in a nominal driving posture. Muscle activation was not involved in either model for any of the presented simulations to remove a potentially confounding factor. Noted in previous work for the case of frontal impact, the kinematic response (peaks of: head and neck rotation, head lag time, as well as head CG displacement, x and z linear acceleration, and y-rotational acceleration) of the modeled head-neck complex was most sensitive to extensor muscle level activation (Dibb, 2011, pg. 127). Cross sections were created using LS-DYNA R7.1.2 (LSTC, Livermore, CA, United States) at the C2 level based on previously published methods (White et al., 2015) with coordinate systems in accordance with SAE J211 convention (Society of Automotive Engineers, 2007). All section, beam elements, and nodes required for Nij calculation were sampled at 10,000 Hz. The Hybrid III ATD reports forces and moments about a pin at the OC-C1 junction of the upper spine. Pertaining to the GHBMC models regional anatomy of the skull-C1 interface, the skull imposes a restriction of rotation in the sagittal plane. Therefore, an analogous cross section location for both the M50-O and M50-OS was determined to be the C2 level rather than OC-C1. Taken from nominal posture, implemented cross section planes, with accompanying cross section sets, are presented in panels A+B and C+D of Figure 1 for the M50-O and M50-OS, respectively. For the M50-O, posterior skin and flesh as well as 3D muscle elements, 1D muscle beams, and the intersection of the osteoligamentous spine were contained within its cross section. For the M50-OS, posterior flesh, skin, 1D muscle elements, as well as 6 DOF beams and 1D discrete springs and dampers, previously discussed, comprised its cross section. Mass of the head above this plane was measured at 4.45 and 4.89 kg for the M50-O and M50-OS, respectively. Variance in mass above the plane between the models is due to the simplifications of the head in the M50-OS. Within the detailed model, anatomical structures are fully meshed for an even distribution of mass. In the M50-OS these structures are not meshed to reduce computational cost. Rather, the simplified model utilizes a mass node at the CG of the analogous parts, which implicitly does not evenly distribute throughout the resulting cavity. All input files for simulation were developed and modified in LS-PrePost, R4.5 (LSTC, Livermore, CA, United States).

Due to the differences in development of the two aforementioned models, matched-pair tests were conducted to analyze differences in computed Nij. Eq. 1 was used to calculate Nij based on tabulated critical intercepts for a 50th percentile male (Eppinger et al., 2000). Due to the constituents of Nij, simulations were developed to induce, primarily, axial tension and compression forces and moments within the sagittal plane. In 44 total simulations, both impact driven (*n* = 30) and inertially driven through whole body simulation via impact or sled cases (*n* = 14) were completed and analyzed.

**FIGURE 2:**
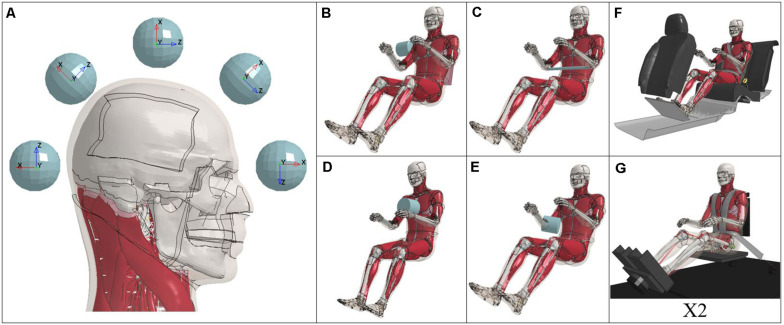
Testing configurations: Impactor **(A)**, rigid hubs **(B–E)**, and full body sleds **(F,G)**.

A rigid spherical impactor, 60 mm in diameter and 9.15 kg drove impact comparisons. These 15 paired simulations (*n* = 30, total) are comprised of 3 impact speeds, 0, 6, and 9 m/s, at 5 impact locations along the mid-sagittal plane (Figure 2A). Impactor locations began anterior to the head center of gravity (CG) (forehead, designated 0°) and rotated around the CG in 45° increments. The impactor was constrained to move in the X (anterior to posterior) direction of its local coordinate system (Figure 2A). This set up allowed for impacts at 0, 45, 90, 135, and 180° within the midsagittal plane to induce a range of flexion/extension and compression/tension forces. For severe impacts on the anterior aspect of the head (0° at 9 m/s and 45° at 6 and 9 m/s), negative volume computational errors of the exterior most hexahedral elements representing the scalp directly beneath the impactor were assigned rigid properties. This change was only made to an area of the scalp on the order of 0.3 m^2^ and would not affect the biomechanics of the simulation. Whole body simulation configuration and occupant position are shown for the M50-O (Figures 2B–G) with rigid impactors shown in gray. These simulations mimicked the experimental testing configurations of previously validated (Vavalle et al., 2013; Hayes et al., 2014) and published rigid hub (*n* = 8) (Figures 2B–E) (Kroell et al., 1971; Viano, 1989; Hardy et al., 2001; Koh et al., 2005) and full body sled pulses (*n* = 6) (Figures 2F,G) (Wismans et al., 1986; Forman et al., 2006). Due to the complexity of two full body testing configurations (Figure 2G), the model versions used for the original simulation development were used in this study (versions 4.5 and 1.8.4 of the M50-O and M50-OS, respectively), although no changes in the neck models were made for these later versions. To remove any potentially confounding factors, fracture within the detailed model was disabled. All simulations were run on the WFU DEAC cluster.

Data was extracted via T-His [Oasys T/HIS 15.0–64 bit (Arup, London, United Kingdom)]. Further processing and filtering at SAE J211 CFC 1000 and 600 for forces and moments, respectively, in accordance with FMVSS regulation (NHTSA, 2008), was conducted in MATLAB (R2014a, MathWorks, Natick, MA, United States). Cross section outputs within the binary output files report forces and moments about the centroid of the cross section. In order to achieve equivalent locations for Nij calculation between models, output data was translated to the centroid of the C2 vertebrae. This was accomplished by application of a transformation matrix to the cross section outputs of the models. Since the forces reported by the cross sections are relative to the desired, local coordinate system, by the principle of transmissibility, forces will not be altered when translated to the C2 CG. Alternatively, the transformation of the reported cross section moment requires a modified moment component based on the forces (Fz and Fx) of the cross section and the distance, or moment arm, to the C2 centroid. While the M50-O model translates the output of the cross section to the C2 CG then sums the appropriate moment correction, the M50-OS model requires an additional step of rotating the beam outputs from the beams local coordinate system to the global coordinate system before undergoing translation. To provide a closer representation of the C2 level in the M50-OS, contributions of the C2–C3 and OC–C1 beams were extracted and their contributions weighted based on relative proximity to the C2 CG. For example, the approximate distances of the OC–C1 and C2–C3 beams from the C2 CG are initially 27.5 and 14.5 mm, respectively. Therefore, the initial contribution of the C2–C3 beam would be roughly twice as much as the OC–C1 beam. The relative contribution of each beam was dynamic throughout each simulation. Nij was calculated at each time point of the simulation.

In order to evaluate trends exhibited in this experiment, *ad hoc* analyses of the head and neck kinematics, as well as the time of Nij, were necessary. Transformed forces (axial force, *F*_*z*_) and moments of each model were plotted for the length of each simulation (Supplementary Figures A3, A4). Comparison of global head CG kinematics (displacement), as well as local head CG kinematics (resultant, linear and angular accelerations), was achieved by a custom Python script. Modifications to the aforementioned script was also used for comparison of timing of maximum Nij and neck angle kinematics. Neck angle was calculated between a vector from the head CG (Frankfurt Plane) coordinate system relative to a vector from the CG of the C7 vertebrae to the midpoint of its anterior aspect in order to consider motion of the entire head-neck complex (Supplementary Figure A5). Neck angle nodal coordinates, linear and angular head accelerations, were each filtered at SAE J211 CFC 1000 in accordance with FMVSS regulation (NHTSA, 2008).

## Results

**FIGURE 4:**
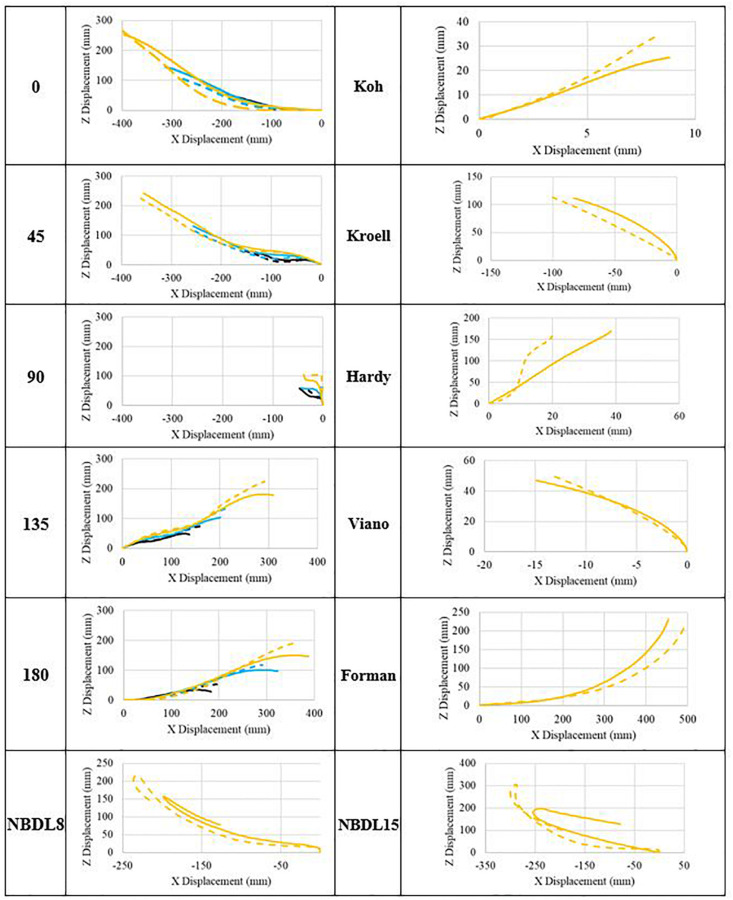
Head CG of M50-O (dashed) and M50-OS (solid) at 3 (blk), 6 (blu), and 9 m/s (yel).

Global head CG kinematics were plotted to identify potential differences resulting from variation in model performance. Traces of *X* relative to *Z* displacements are plotted for each test configuration based on SAE J211 convention (Society of Automotive Engineers, 2007; Figure 4). Displacement of the test buck was subtracted from the motion of the head CG in order to isolate its motion in the cases of Forman, NBDL8, and NBDL15. In terms of *X* displacement, generally speaking, the models have similar kinematic response to impact for a majority of cases. *Z* displacement indicates that the head motion of the M50-OS is relatively more than the M50-O in instances of neck extension, while the opposite trend is apparent for neck flexion. Scale of the axes is of particular note in the interpretation of kinematics shown in Figure 4. At a glance, the Hardy plot appear to be the most divergent, however, the magnitude of this difference is marginal relative to the head impact trials which overall exhibited a greater magnitude of displacement across all trials.

*Ad hoc* analysis conducted to obtain the time history of neck angle between the Frankfurt Plane and C7 vertebrae was used to further elucidate factors associated with varied kinematic response in the models. Time history traces of change in neck angle are found in Supplementary Figure A5 of the Appendix. Across all trials, the maximum neck extension and flexion angles for both models occurs during the 9 m/s, 0° impact and NBDL15 simulations, respectively. For the M50-O, maximum extension and flexion were found to be 47.6 and −47.6°, respectively, while the M50-OS reported maximums of 43.2 and −52.4°. It should be noted that maximum flexion of both models was limited due to the contact of the chin to the chest. Pertaining to impact simulations, qualitative evaluation of neck angle shows a greater compliance of the M50-OS head-neck complex in extension for a majority of the time history. For cases of flexion (135 and 180° impact locations), both models initially trend in a similar fashion upon contact while the M50-O diverges further into flexion later in the time history.

Time history traces of resultant linear and angular head acceleration for each trial are provided in the appendix (Supplementary Figures A1, A2). For each of these figures, impact driven simulations are plotted from 0 to 20 ms while full body inertial loading cases (Figures 2B–G) are shown for the entirety of their simulation. No time offset has been applied to the impact driven simulations between 3, 6, and 9 m/s trials. Therefore, the delay in time to peak acceleration at different speeds is an intended consequence of the initial spacing between the scalp and impactor. In the case of both linear and angular acceleration, whole body inertial loads exhibit similar trends in both magnitude and temporal domain which is not evident in the response of the head to impact driven testing configurations. Inertial loading cases induced linear and rotational head accelerations that were lesser in magnitude compared to impact simulations. To this point, additional consideration should be taken when comparing plots of impact and inertial loading conditions. Overall, the detailed and simplified models reported higher peak angular and linear accelerations, respectively, relative to one another in a majority of cases.

Force and moment time histories for the entirety of each simulation are also provided in the appendix (Supplementary Figures A3, A4). The range of forces and moments exhibited by the detailed model are approximately [1.9, −2.9 kN] and [29, −27 N^∗^m]. In a majority of cases, the simplified model reported higher axial forces compared to the detailed. Specifically in the impact driven simulations, the time to peak force reported by the M50-OS is achieved earlier, with a more pronounced rise and fall. This trend is not replicated when the models are matched for instances of full body, inertial loading. In terms of peak moment, the simplified model generally reported lower values. In the case of lateral shoulder impact [Koh (Figure 2B)], the simplified model is in disagreement with the detailed in terms of whether the neck is being forced into extension or flexion. As it has been previously noted within the literature (Jones, 2019), the delineation of a specific trend which relates the moments of the upper neck region between the models remains unclear, which is consistent with the present findings.

**FIGURE 3:**
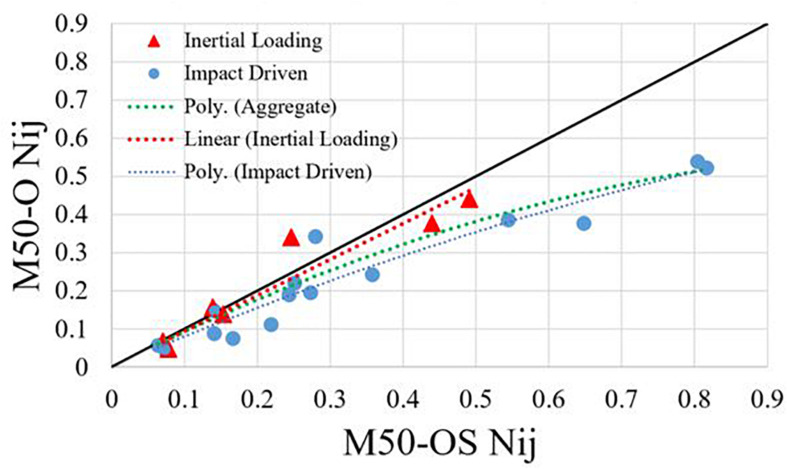
Cross-plot of Nij for each match paired simulation between M50-O and M50-OS.

While Nij was calculated at each time point of the simulation, the maximum of the calculated values was taken to be the corresponding value for comparison. A cross plot of the results from the match-paired simulations supports that the M50-OS reports greater maximum values of Nij compared to the M50-O (Figure 3). A slope approaching unity (displayed as a black trend line) would indicate that across all samples, the models exhibit a linearly correlated measure. Blue circles represent data points from the impactor simulations while red triangles represent rigid hub and sled simulations. Of the red data points, the three highest Nij values of the set correspond to sled tests. Accounting for all data points, the comparison of Nij between models exhibits a second-order polynomial fit with relatively low variance (*R*^2^ = 0.885). As it is evident that differences in reported Nij may be associated with variance in applied boundary conditions (impact driven or inertial loading scenarios), Eqs 2–4 below present the trend lines in Figure 3: Eq. 2 = Poly. (Aggregate) (*R*^2^ = 0.885), Eq. 3 = Linear (Inertial Loading) (*R*^2^ = 0.900), and Eq. 4 = Poly. (Impact Driven) (*R*^2^ = 0.915), each of which may be used as correlates between M50-OS and M50-O Nij values when applicable. While the inertial loading trend line appears linear and approaching unity, this is not the case in impact driven trials. Greater variance in reported Nij values between the models occurs during the higher loading rates impact driven trials. Therefore, a second order polynomial fit is justifiable when describing this relationship.

(2) $$Y=-0.4061\,X^{2}+0.9666\,X$$

(3) Y= 0.9429⁢X

(4) $$Y=-0.229\,X^{2}+0.8226\,X$$

## Discussion

Differences in head and neck kinematics aid in delineating the mechanism of divergence between the models. The M50-OS model exhibited a greater magnitude of change in neck angle at the instance of impact for the 0, 45, and 90° trials. This is supported by the early divergence in head CG kinematics while maintaining similar end positions. For changes in neck angle during the 135 and 180° trials, the M50-O and M50-OS are qualitatively in close agreement at the instance of impact but deviate as the M50-O neck continues further into flexion (Supplementary Figure A5). This finding is also supported by the trend in head CG kinematics exhibited in Figure 4 for the 135 and 180° cases. In these aforementioned cases, the head of the M50-OS does not continue further into the +Z direction in the same way that the M50-O does. Dibb et al., whose research provided the foundational work related to the design of the M50-OS neck architecture, noted that secant bending stiffness of the simplified neck is only sensitive to parameters of the simplified muscle elements (Dibb, 2011, pg. 130). Within the previous work, sensitivity analyses were conducted on select parameters (head geometry, intervertebral joints, muscle properties, and muscle activation) for a simplified, isolated head and neck model containing muscle and osteoligamentous spine while also employing active musculature. Three analyses were conducted within Dibb’s work of an adult model: a simplified condition, relative to the present study, of the NBDL15 (Figure 2G) trial, pure tension experiment, and flexion and extension testing via pendulum. Results of the NBDL15 trial between the present work and that of Dibb et al. show that the current study exhibits increased head displacement and acceleration [consistent with their findings (Dibb, 2011, pg. 127)], marginally reduced neck rotation (roughly 1.5 degrees less, due to the contact of the head to the chest in the present study), increased forces, and decreased moments. The latter portion of this comparison (force and moment relationship) could be attributed to the lack of muscle activation within the current work. Overall, the comparison of the present work to that of Dibb in the case of the NBDL15 trial are in line with the findings of the previous researchers.

In the case of NBDL15, Dibb et al. noted several influential parameters affecting model response, in which response is taken to be OC2 peak tension force, OC2 peak extension moment, OC2 Nij, and neck bending stiffness. Aside from initial acceleration within the boundary condition, the most significant parameters studied were extensor activation level and reflex time, muscle attachment location, PCSA, max isometric stress, as well as flexion and extension stiffness parameters of the osteoligamentous joint space (Dibb, 2011, pg. 132). Properties related to head geometry were found to affect the peak CG displacement in the X direction alone (Dibb, 2011, pg. 129). Muscle parameters (to name a few, in order of significance, x attachment, max isometric stress, and PCSA) were the only parameters found to influence response of the flexion and extension pendulum testing (Dibb, 2011, pg. 165). Lastly, during pure tension application, the most significantly influential parameters on model response were tensile (Fz) stiffness of the intervertebral joints and the following muscle parameters: PCSA, z attachment, passive stiffness (Dibb, 2011, pg. 188). In this testing regime, the researchers also noted that the use of single or multi-segment muscles resulted in significant differences in neck tolerance, segment loads, and tensile stiffness. When modeling neck muscles as multiple segments, intermediate points which attach at several anatomically accurate regions along the muscles length were found to significantly impact muscle loading line of action in the case of flexion (Dibb, 2011, pg. 87). As neck muscles are modeled as single strands in the M50-OS, the loading line of action between the M50-O and M50-OS will differ from one another.

Therefore, differences in the anatomical architecture of the M50-O and M50-OS necks are relevant to consider in the interpretation of the results of the present study. Considering that active musculature has been excluded within the current study as a potentially confounding variable, the remaining parameters that have been identified as influential above include: muscle attachment location (origin and insertion), employment of single or multi-segment muscle modeling approach, muscle PCSA, head geometry, and stiffness parameters of the osteoligamentous joint space. In summation, the work of Dibb et al. specifically highlights select parameters that are most influential on model response. As a consequence of these factors, there is likely to be divergence in head and neck kinematics, and ultimately, reported forces and moments.

The similarities in neck rotation of the simplified and detailed models are accompanied with the caveat that neck flexion was restricted by contact of the inferior portion of the head to the chest. In terms of extension only, the change in head-neck angle of the M50-O closely mimics that of the M50-OS during the initial time history (Supplementary Figure A5). These trends may also be understood in the context of the linear and angular acceleration of the head CG of each model. In a majority of trials, the M50-O achieves higher values of angular acceleration which is consistent with the greater moments, and change in neck angle during extension, reported by the M50-O. However, the relative increase in head acceleration kinematics varies between trials. In the case of impact trials, the M50-OS generally reports higher linear acceleration. This differs from the whole body trials where max acceleration is greater for the M50-O and occurs later in the time history. The localized mass of the M50-OS head, as opposed to the greater distribution in the M50-O, would directly impact the kinematics of the head CG between the models. As previously described, the conversion of the detailed model to its simplified counterpart results in implicit differences that have previously been identified as significant influences on model response (Dibb, 2011). While Dibb concentrated efforts on a single model in large parametric studies, in comparison, the conclusions of this work must be understood with the added variability of modeling differences.

When comparing force and moment in an equivalent manner to the Nij cross plot, a correlate for reported forces at Nij was observed and provided by Eq. 5 below (*R*^2^ = 0.8237). As has been previously noted by Jones (2019) sagittal moment was not found to correlate between the models used in this study (Jones, 2019). Time history of sagittal moment has been graphically represented in Supplementary Figure A4 of the appendix. Of the influential parameters listed above, individual factors cannot be isolated as the cause of this trend. Due to the natural lordosis of the spine, variance in kinematics is likely to illicit difference in the upper neck axial force and sagittal moment reported by each model due to the result of a complex loading scenario. To offer one potential explanation, when considering the effects of parameters within the intervertebral joint space (tension, flexion, and extension), greater compliance of the M50-OS neck may result in greater forces and reduced moments as the 6DOF beams between the vertebrae are being compressed/extended to a greater extent compared to analogous structures of the M50-O.

(5) y=0.6246⁢x

The relation of Nij between the models is intended to be displayed as a consequence of the previously described kinematics of the head-neck region. It may be more appropriate to model the overall trend between M50-O and M50-OS Nij calculations as a second order polynomial correlation due to the larger sample of impact driven trials. Within the impact driven trials, greater divergence in Nij values reported by the M50-OS during cases of high impact loading appear to drive this trend. On the other hand, during instances of inertial loading, the models present with similar reported Nij values evident by Eq. 3 above. In cases of head impact (Figure 2A), the max Nij was determined to be around the time of impact for both models. Cases of inertial loading warrant stratification. The sled simulations (Figures 2F,G) achieved the greatest Nij around the time of maximum head excursion. During rigid hub impacts (Figures 2B–E), maximum Nij was calculated further into the simulation due to the lag in head-neck motion and subsequent head rotation. Across all trials, the max Nij was generally calculated around similar time points of the simulation between models. The greater magnitude of Nij calculated by the –OS model may be further explained by differences in head and neck kinematics. While we have introduced separate regression functions for inertial and impact loading, the important question of why these responses diverge remains. We believe that it is possible to eliminate certain factors as candidates for this behavior. Relative to the passive property of the muscle, the insertion and origin points are unlikely to lead to such differences since they are all within millimeters of each other on the respective spines of both models and the bones of each model are identical meshes and shapes. Furthermore, we do not see appreciable differences in PCSA between the two models (e.g., Introduction). The only consideration relative to musculature is that the M50-O 1-D muscles utilize multiple elements with intermediate nodes whereas the muscles in the M50-OS are represented by a single beam element. Regarding head mass, the brain model of the M50-O has an evenly distributed mass where the M50-OS relies on a point mass at the CG of the equivalent anatomy. It should be noted that the mass of the components which constitute the head and neck of each model are approximately equivalent to one another. Outside of the neck muscle, as noted in the introduction, the M50-OS total number of elements, excluding the osteoligamenous spine, is ∼3,000 whereas the analogous number of elements in the M50-O is ∼173,000. This basic accounting of the element number (and therefore element size since the volume are the same) would signal a potential for differing behavior. Recall the M50-OS model was designed to run on the order of 30–40 times faster than the M50-O and therefore has courser elements. While we do not have enough data to assign causation to any of these factors, it is possible that they are correlated to the difference. Regarding what leads to the specific difference between the inertial and impact loading, it is currently unclear if the implementation of neck anatomical architecture from M50-O to M50-OS, or the subsequent biomechanical response of this implementation, is a greater contributor to divergence in behavior.

While head kinematics track reasonably well between the two models (Figure 4), it is important to also consider injury prediction via Nij. Applying the polynomial correction above narrows the observed deviation of the M50-OS Nij value from the M50-O value. The next step therefore is to evaluate the difference in injury prediction before and after this correction. This may be evaluated by using injury risk functions to correlate Nij to risk of AIS 2+, 3+, 4+ and 5+ injuries (Eppinger et al., 2000). Prior to using the correlation, M50-OS Nij values lead to an average percent difference of 12.0% from M50-O across all injury severities. This difference was reduced to 5.0% after the *ad hoc* correlation was implemented. Therefore, with the derived correlate the M50-OS is able to more accurately approximate AIS 2–5 injury risks within 5.0% of the M50-O.

In determining critical intercepts for Nij, researchers have attempted to design experimental set ups which allow for application of only one of the following loading modes: compression, tension, flexion, or extension. For example, in the determination of the compression critical intercept, Pintar et al., 1998 found intercepts similar to the Hybrid III ATD when the natural lordosis of the cervical spine was removed (Pintar et al., 1998). This finding was also supported by Yoganandan et al. (1991). In its natural position, however, force ranging from 1.78–4.45 kN were observed to cause failures due to compression-flexion (Sances et al., 1982). These initial findings are further confounded as Panjabi et al. (1991) identified an average load of 2.9 ± 0.6 kN for both neutral and straightened skull to C3 functional units of which, 10 out of 13 specimen exhibited Jefferson like fracture. Coupled with this, multiple researchers have also noted the sensitivity of injury and force measures to the positioning of the neck (Maiman et al., 1983; Mcelhaney et al., 1983). A similar gap in knowledge exists for the investigation of tension. In this case, most suggested values are based around the work of Sances et al. (1982) which demonstrated a mean load to produce ligamentous damage of 1.5 ± 0.5 kN. As this value does not incorporate passive muscle tone which would allow for greater forces to be sustained, a more exact measure is unknown due to intrapersonal differences and the natural curvature of the spine. While Nij requires intercepts based on pure compression, tension, flexion, and extension, this task is especially difficult to investigate experimentally due to the complex loading mechanics attributed to the lordosis of the cervical spine.

In summation, the correlation (*R*^2^ = 0.885) that is seen across all test configurations is an indication that the identified fit would provide a close approximation of a matched M50-O simulation for calculated Nij and, ultimately, injury risk. Two correlates have been delineated from the overall fit in order to provide loading specific transfer equations. Due to the fewer trials of inertial loading carried out in this body of work, future studies may compare the M50-O and M50-OS head-neck complex under a larger variety of inertial loading scenarios. The number of trials used was substantiated by the use of the jackknife resampling method to estimate variance and bias [resampling data (n–1)]. For example, the coefficients of Eq. 2 above were calculated for all trials except the 0–3 case, the fit was recorded, 0–3 was placed back into the selected data, 0–6 was removed, the fit was again determined, and so on. Standard error for each coefficient was found to differ by <2% with an average correlation coefficient of 0.94. This analysis showed that the *R*^2^ value did not change by more than 4% and the coefficients are within the 95% confidence interval.

With testing configurations primarily in the AP direction, results may lack extensibility to cases of lateral loading. Across all trials the M50-O exhibited Nij values from 0.0454 to 0.5389. There is an inherent limitation in this work through the use of published critical intercepts and injury risk curves. For example, there is >0% risk of injury for a Nij value of 0 due to the statistical regression method that was used in their development. Within the specific range of Nij values tested, the risk curves for AIS3+ and AIS4+ injuries intersect. Despite this limitation, correction of critical intercept or AIS risk curves would impact the prediction of each model in an equivalent manner meaning that the trends derived in this work would still remain valid. Additionally, while measures for force and moment are taken from the occipital-condyle portion of an ATD’s neck section, the C1–3 levels of the GHBMC M50-O model has previously been shown to have a marginal difference in terms of axial force and bending moment time history and magnitude (White et al., 2015). Future work utilizing the M50-O should attempt to determine how appropriate the defined critical intercepts are for finite element human body models. Given the background for determination of these intercepts in ATD’s and cadavers, more research is needed before they can be applied to GHBMC models.

## Conclusion

The calculated Nij between the M50-O and M50-OS models were comparable with strong correlation (*R*^2^ = 0.8848), consequent to application of an *ad hoc* correlate. This finding is based on a test matrix comprised of matched-pair impactor, rigid hub, and sled simulations and were predominantly in the sagittal plane. Despite this correlation, kinematics of the head CG showed deviations at large displacements, particularly in the Z direction. The relative magnitude of Nij constituents was also shown to differ. It is likely that the increase in calculated Nij and greater resistance to flexion by the M50-OS neck after insult via impact may be attributed to differences in modeling approach. A variety of factors encompass this point and should be taken in two parts, (1) implementation and (2) biomechanical response of this implementation from the M50-O to the M50-OS. A clear cause of the exhibited differences cannot be delineated at this time, rather a variety of factors have been explored and may be summarized to: muscle modeling parameters (muscle attachment site, PCSA, intermediate attachment sites), properties of the intervertebral joint space (tension, flexion, and extension), distribution of mass as well as geometry of the head may be at play. Future work should evaluate the established trend for inertial loading scenarios through additional loading cases. Consequently, the applicability of defined critical intercepts for Nij as they relate to the GHBMC human body models may also be undertaken.

## Data Availability Statement

All datasets generated for this study are included in the article/Supplementary Material.

## Author Contributions

DJ implemented C2 neck planes into both M50 models, created rigid head impactor simulations, post processed and analyzed simulations between the models, wrote the custom python script for head kinematics as well as component comparison, improved MATLAB processing of M50-OS NIJ, wrote the manuscript. BK wrote the custom T-His and MATLAB scripts for the post-processing and determination of NIJ in both M50-O and M50-OS models. FG provided concept for the study, advisement of testing configuration and data collection as well as assisting in manuscript review and interpretation of results. All authors contributed to the article and approved the submitted version.

## Conflict of Interest

FG is a member of Elemance, LLC., which distributes academic and commercial licenses for the use of GHBMC-owned computational human body models. The remaining authors declare that the research was conducted in the absence of any commercial or financial relationships that could be construed as a potential conflict of interest.

## References

[B1] Association for the Advancement of Automotive Medicine (2018). *Abbreviated Injury Scale: 2015 Revision*, 6 Edn, Chicago, IL: Association for the Advancement of Automotive Medicine.

[B2] Barbat S. Fu Y. Zhan Z. Yang R. J. Gehre C. (2013). *Objective Rating Metric for Dynamic Systems.* Seoul: Enhanced Safety of Vehicles Seoul.

[B3] Bruneau D. Cronin D. (2019). Assessment of head kinematics for bare head and helmeted impacts comparing an ATD and a detailed head and neck model with active musculature. *J. Biomechan. Eng.* 142:667 10.1115/1.4043667

[B4] Decker W. Koya B. Davis M. L. Gayzik F. S. (2017). Modular use of human body models of varying levels of complexity: validation of head kinematics. *Traffic Injury Prevent.* 18 S155–S160. 10.1080/15389588.2017.1315637 28414545

[B5] Dibb A. (2011). *Pediatric Head and Neck Dynamic Response: A Computational Study.* Dissertation, Duke University, Durham Available online at: http://dukespace.lib.duke.edu/dspace/handle/10161/3811

[B6] Eppinger R. Sun E. Saul R. Kleinberger M. Sun E. Eppinger R. (2000). *Supplement: Development of Improved Injury Criteria for the Assessment of Advanced Automotive Restraint Systems II.* Washington, DC: NHTSA.

[B7] Forman J. Lessley D. Kent R. (2006). Whole-body kinematics and dynamic response of restrained PMHS in frontal sled tests. *Stapp. Car Crash J.* 50 299–336. 17311169 10.4271/2006-22-0013

[B8] Gaewsky J. P. Jones D. A. Ye X. Koya B. McNamara K. P. Gayzik F. S. (2019). Modeling human volunteers in multidirectional, Uni-axial sled tests using a finite element human body model. *Ann. Biomed. Eng.* 47 487–511. 10.1007/s10439-018-02147-3 30311040

[B9] Gayzik F. S. Koya B. Davis M. L. (2018). A preliminary study of human model head and neck response to frontal loading in nontraditional occupant seating configurations. *Traffic Injury Prevent.* 19 S183–S186. 10.1080/15389588.2018.1426915 29584505

[B10] Gayzik F. S. Moreno D. P. Geer C. P. Wuertzer S. D. Martin R. S. Stitzel J. D. (2011). Development of a full body CAD dataset for computational modeling: a multi-modality approach. *Ann. Biomed. Eng.* 39 2568–2583. 10.1007/s10439-011-0359-5 21785882

[B11] Gehre C. Gades H. Wernicke P. (2009). *Objective Rating of Signals Using Test and Simulation Responses.* Washington, DC: National Highway Traffic Safety Administration.

[B12] Goldberg W. Mueller C. Panacek E. Tigges S. Hoffman J. R. Mower W. R. (2001). Distribution and patterns of blunt traumatic cervical spine injury. *Ann. Emerg. Med.* 38 17–21. 10.1067/MEM.2001.116150 11423806

[B13] Hardy W. N. Schneider L. W. Rouhana S. W. (2001). Abdominal impact response to rigid-bar, seatbelt, and airbag loading. *SAE Techn. Pap.* 45 1–32. 10.4271/2001-22-0001 17458738

[B14] Hayes A. R. Vavalle N. A. Moreno D. P. Stitzel J. D. Gayzik F. S. (2014). Validation of simulated chestband data in frontal and lateral loading using a human body finite element model. *Traffic Injury Prevent.* 15 181–186. 10.1080/15389588.2013.799278 24345021

[B15] Jones D. A. (2019). *Head and Neck Injury Risk Predicted by Finite Element ATDs and Human Body Models in the Aerospace Landing Environment.* Dissertation, Wake Forest University, Winston-Salem.

[B16] Koh S. Cavanaugh J. M. Mason M. J. Petersen S. A. Marth D. R. Rouhana S. W. (2005). Shoulder response characteristics and injury due to lateral glenohumeral joint impacts. *SAE Techn. Pap.* 49 291–322. 10.4271/2000-01-SC18 17096279

[B17] Kroell C. K. Schneider D. C. Nahum A. M. (1971). Impact tolerance and response of the human thorax. *SAE Techn. Pap.* 1971:710851. 10.4271/710851 710851

[B18] Maiman D. J. Sances A. Myklebust J. B. Larson S. J. Houterman C. Chilbert M. (1983). Compression injuries of the cervical spine: a biomechanical analysis. *Neurosurgery* 13 254–260. 10.1227/00006123-198309000-00007 6621839

[B19] Mcelhaney J. H. Paver J. G. Mccrackin H. J. Maret G. (1983). Cervical spine compression responses. *Transactions* 92 831406–831830.

[B20] Mertz H. J. Driscoll G. D. Lenox J. B. (1987). Responses of animals exposed to deployment of various passenger inflatable restraint system concepts for a variety of collision severities and Animal positions. *Paper Presented at SAE PT-31, Passenger Car Inflatable Restraint Systems: A Compendium of Published Safety Resear, SAE Technical Papers*, Vol. 1(Warrendale, PA: SAE International), 215–231.

[B21] Mertz H. J. Hodgson V. R. Thomas L. M. Nyquist G. W. (1978). An assessment of compressive neck loads under injury-producing conditions. *Phys. Sports Med.* 6 95–106. 10.1080/00913847.1978.11948406 29256708

[B22] Mertz H. J. Patrick L. M. (1971). “Strength and response of the human neck. SAE paper # 710855,” in *Proceedings of the 15th Stapp Car Crash Conference*, Coronado, CA.

[B23] NHTSA (2008). *Federal Motor Vehicle Safety Standards; Occupant Crash Protection. 49 CFR Part 571 Section 208.* Washington, DC: NHTSA.

[B24] Nightingale R. W. Doherty B. J. Myers B. S. McElhaney J. H. Richardson W. J. (1991). “Influence of end condition on human cervical spine injury mechanisms,” in *Proceedings - Society of Automotive Engineers*, San Diego, CA.

[B25] Nyquist G. W. Begman P. C. King A. I. Mertz H. J. (1980). Correlation of field injuries and GM hybrid III dummy responses for lap-shoulder belt restraint. *J. Biomechan. Eng.* 102 103–109. 10.1115/1.3138204 7412232

[B26] Panjabi M. M. Oda T. Crisco J. J. Oxland T. R. Katz L. Nolte L. P. (1991). Experimental study of atlas injuries I: biomechanical analysis of their mechanisms and fracture patterns. *Spine* 16 S460–S465. 10.1097/00007632-199110001-00001 1801253

[B27] Passias P. G. Poorman G. W. Segreto F. A. Jalai C. M. Horn S. R. Bortz C. A. (2018). Traumatic fractures of the cervical spine: analysis of changes in incidence, cause, concurrent injuries, and complications among 488,262 patients from 2005 to 2013. *World Neurosurg.* 110 e427–e437. 10.1016/J.WNEU.2017.11.011 29138069

[B28] Pintar F. A. Yoganandan N. Voo L. (1998). Effect of age and loading rate on human cervical spine injury threshold. *Spine* 23 1957–1962. 10.1097/00007632-199809150-00007 9779527

[B29] Prasad P. Daniel R. P. (1984). A biomechanical analysis of head, neck, and torso injuries to child surrogates due to sudden torso acceleration. *SAE Techn. Pap*. 23 784–799. 10.4271/841656 841656

[B30] Society of Automotive Engineers (2007). *SAE J211/1 – Instrumentation for Impact Test-Part 1 – Electronic Instrumentation*. Harrisburg, PA: Warrendale.

[B31] Sances A. J. Yoganandan N. Pintar F. A. Kumaresan S. Walsh P. R. (1982). “Impact biodynamics of human skull fracture,” in *Proceedings of the AGARD Conference on Injury Prevention and Cost*, Koln.

[B32] Schap J. M. Koya B. Gayzik F. S. (2019). Objective evaluation of whole body kinematics in a simulated, restrained frontal impact. *Ann. Biomed. Eng.* 47 512–523. 10.1007/s10439-018-02180-2 30523467

[B33] Schwartz D. Guleyupoglu B. Koya B. Stitzel J. D. Gayzik F. S. (2015). Development of a computationally efficient full human body finite element model. *Traffic Injury Prevent.* 16 S49–S56. 10.1080/15389588.2015.1021418 26027975

[B34] Shateri H. Cronin D. S. (2015). Out-of-position rear impact tissue-level investigation using detailed finite element neck model. *Traffic Injury Prevent.* 16 698–708. 10.1080/15389588.2014.1003551 25664486

[B35] Stephan K. Huber S. Häberle S. Kanz K.-G. Bühren V. van Griensven M. (2015). Spinal cord injury—incidence, prognosis, and outcome: an analysis of the TraumaRegister DGU. *Spine J.* 15 1994–2001. 10.1016/J.SPINEE.2015.04.041 25939671

[B36] Svensson M. Lövsund P. (1992). “A dummy for rear-end collisions: development and validation of a new dummy neck,” in *Proceedings Of The 1992 International Ircobi Conference On The Biomechanics Of Impacts*, New York, NY.

[B37] Vavalle N. A. Moreno D. P. Rhyne A. C. Stitzel J. D. Scott Gayzik F. (2013). Lateral impact validation of a geometrically accurate full body finite element model for blunt injury prediction. *Ann. Biomed. Eng.* 41:497. 10.1007/s10439-012-0684-3 23135331

[B38] Viano D. C. (1989). Biomechanical responses and injuries in blunt lateral impact. *J. Passeng. Cars* 98 1690–1719.

[B39] White N. A. Moreno D. P. Gayzik F. S. Stitzel J. D. (2015). Cross-sectional neck response of a total human body FE model during simulated frontal and side automobile impacts. *Comput. Methods Biomech. Biomed. Eng.* 18 293–315. 10.1080/10255842.2013.792918 23930772

[B40] Wismans J. Philippens M. Van Oorschot E. Kallieris D. Mattern R. (1987). “Comparison of human volunteer and cadaver head-neck response in frontal flexion,” in *Proceedings of the 31st Stapp Car Crash Conference*, Berlin.

[B41] Wismans J. Van Oorschot H. Woltring H. J. (1986). Omni-directional human head-neck response. *SAE Transact.* 95 819–837.

[B42] Yoganandan N. Pintar F. A. Sances A. Reinartz J. Larson S. J. (1991). Strength and kinematic response of dynamic cervical spine injuries. *Spine* 16(10 Suppl.), S511–S517. 10.1097/00007632-199110001-00011 1801263

[B43] Yoganandan N. Sances A. Pintar F. (1989). Biomechanical evaluation of the axial compressive responses of the human cadaveric and manikin necks. *J. Biomechan. Eng.* 111 250–255. 10.1115/1.3168374 2779191

