# Supplementary material for: Comparison of Neck Injury Criteria Values Across Human Body Models of Varying Complexity
Source: Front Bioeng Biotechnol. 2020 Aug 18;8:985. doi: 10.3389/fbioe.2020.00985 (PMC7462006; doi:10.3389/fbioe.2020.00985)
Supplement: Supplementary file 1 [file Image_1.pdf]

**0**	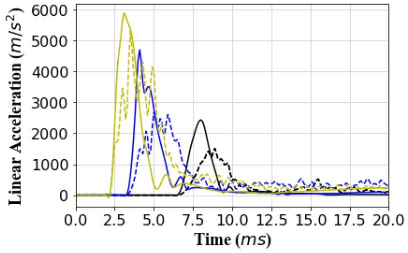	**Koh (Fig. 2B)**	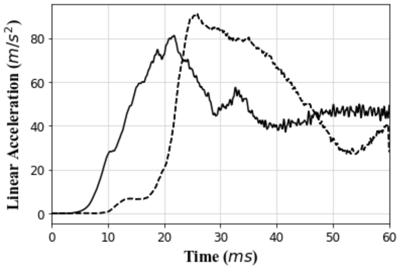
**45**	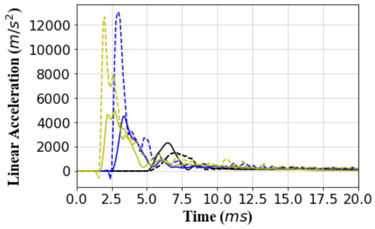	**Kroell (Fig. 2D)**	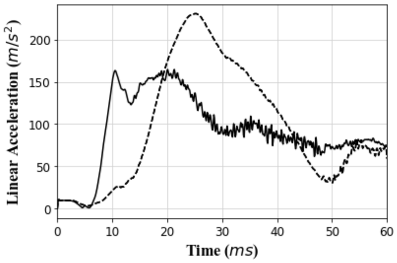
**90**	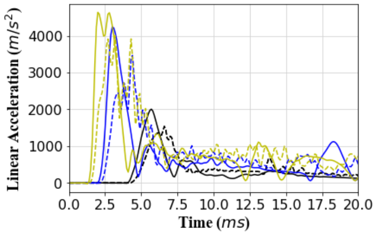	**Hardy (Fig. 2C)**	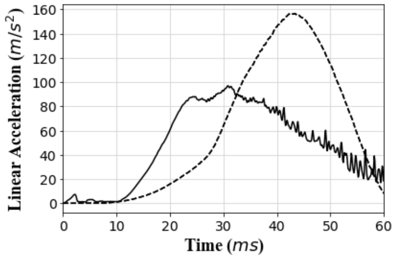
**135**	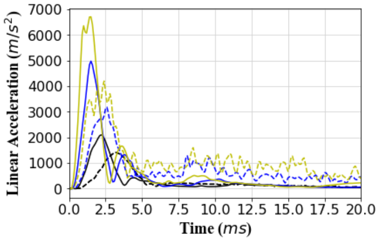	**Viano (Fig. 2E)**	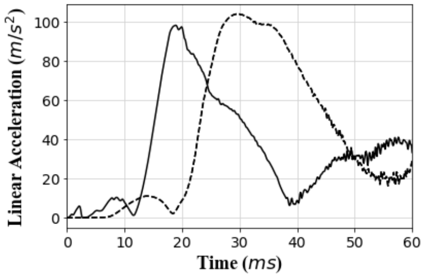
**180**	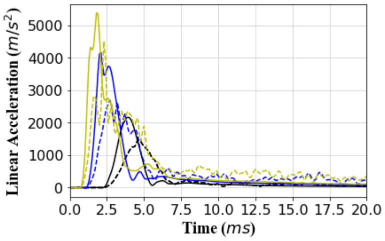	**Forman (Fig. 2F)**	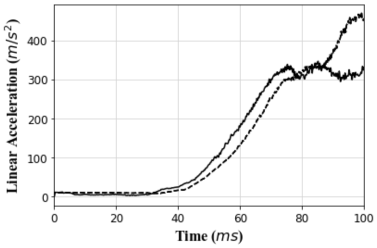
**NBDL8 (Fig. 2G)**	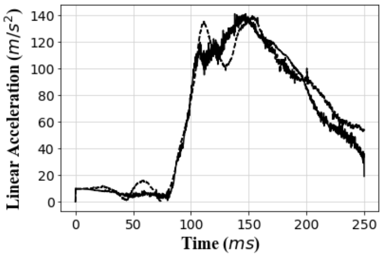	**NBDL15 (Fig. 2G)**	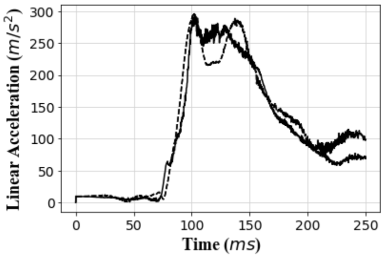

Figure A1: Linear Acceleration of Head CG. M50-O (dashed), M50-OS (solid) at 3 (blk), 6 (blu), and 9 m/s (yel)

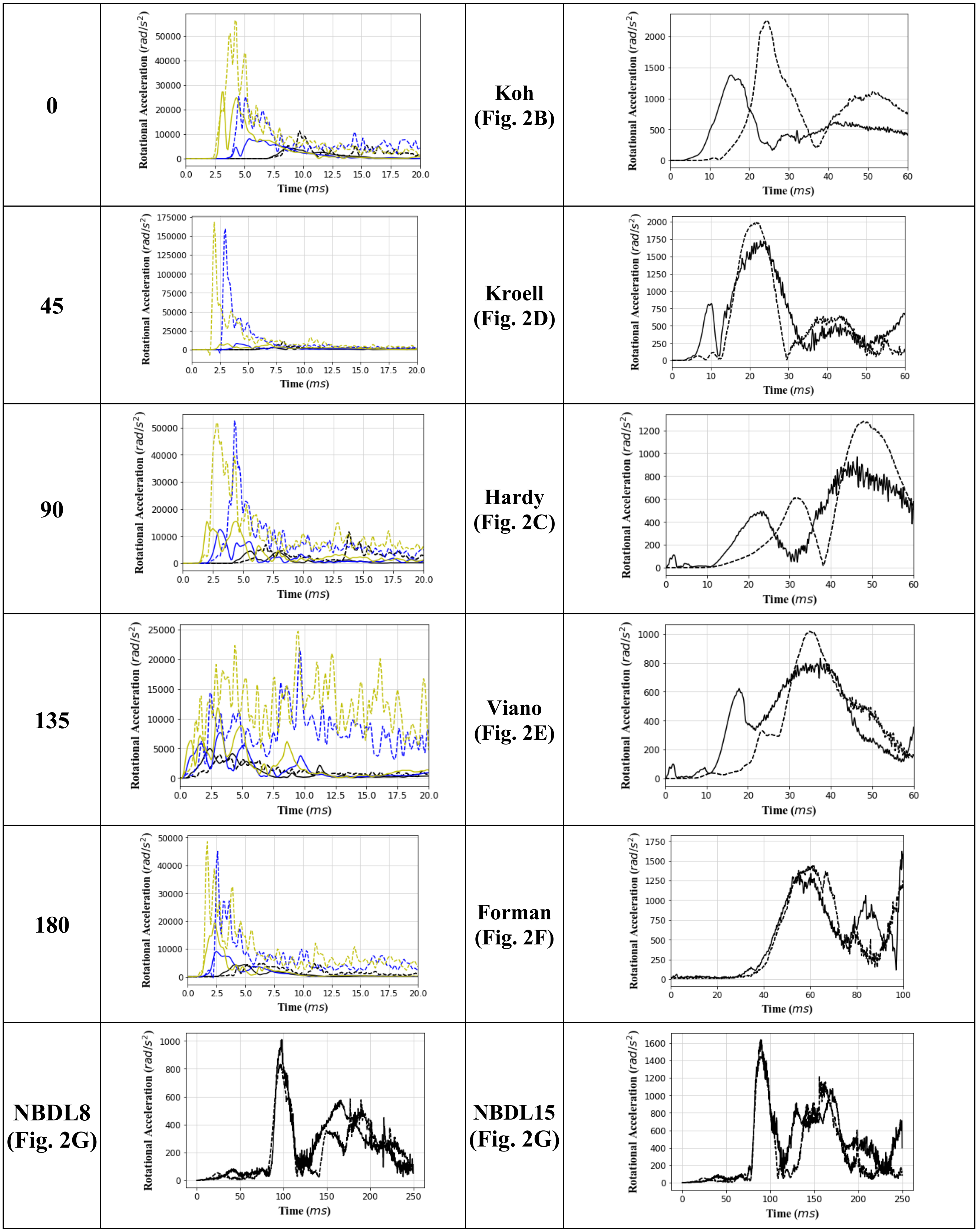

Figure A2: Angular Acceleration of Head CG. M50-O (dashed), M50-OS (solid) at 3 (blk), 6 (blu), and 9 m/s (yel)

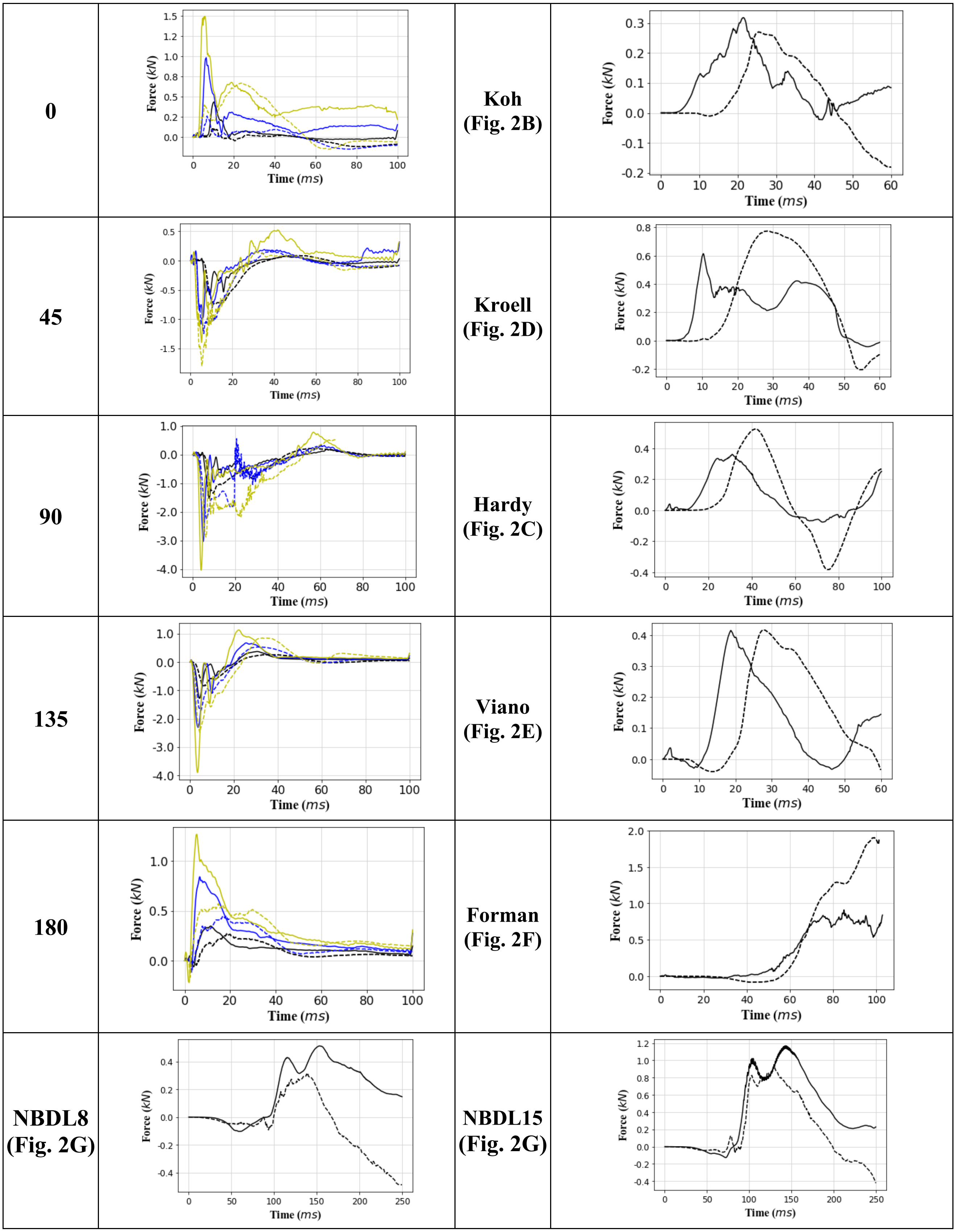

Figure A3: Neck Force. M50-O (dashed), M50-OS (solid) at 3 (blk), 6 (blu), and 9 m/s (yel)

**0**	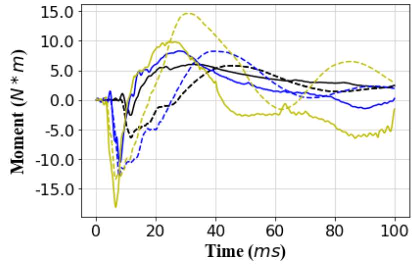	**Koh (Fig. 2B)**	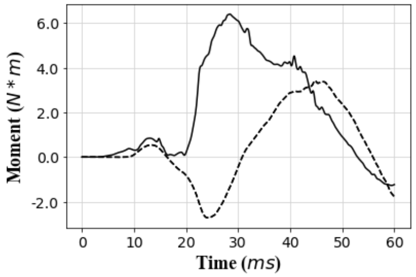
**45**	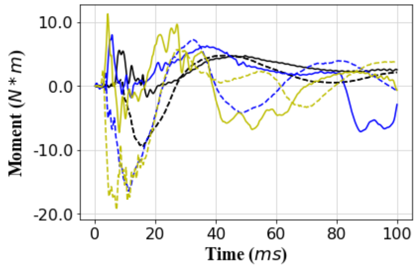	**Kroell (Fig. 2D)**	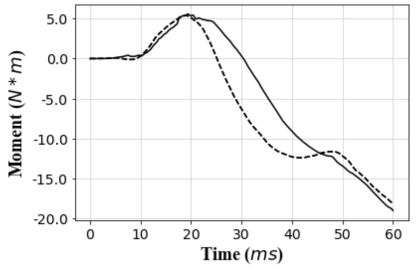
**90**	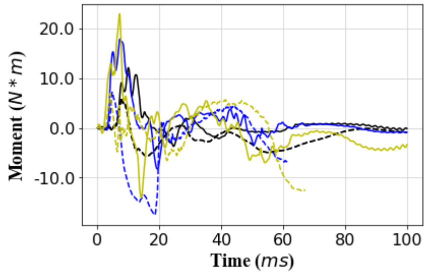	**Hardy (Fig. 2C)**	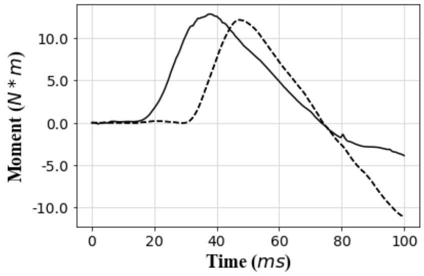
**135**	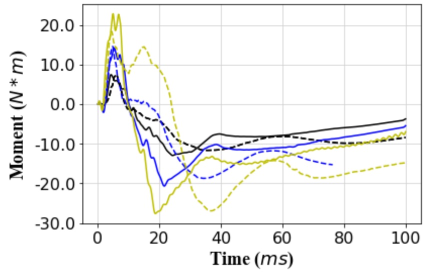	**Viano (Fig. 2E)**	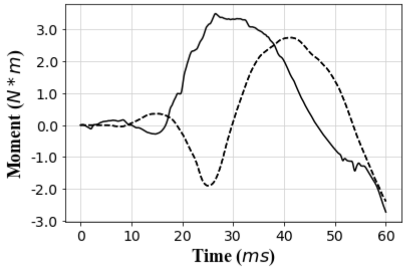
**180**	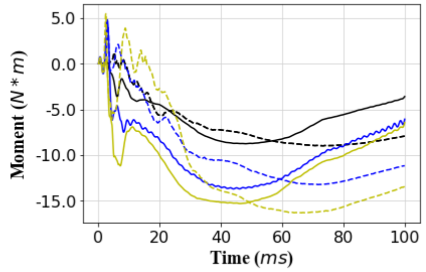	**Forman (Fig. 2F)**	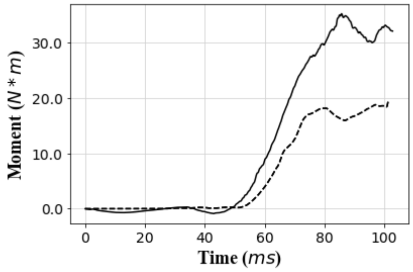
**NBDL8 (Fig. 2G)**	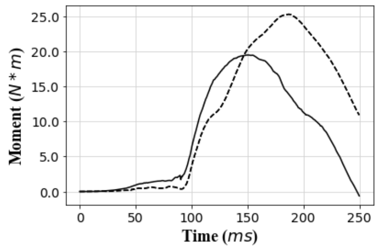	**NBDL15 (Fig. 2G)**	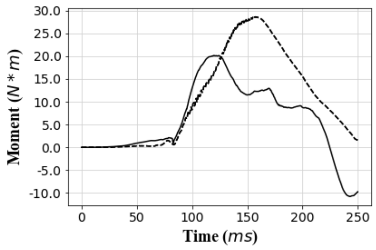

Figure A4: Neck Moment. M50-O (dashed), M50-OS (solid) at 3 (blk), 6 (blu), and 9 m/s (yel)

0

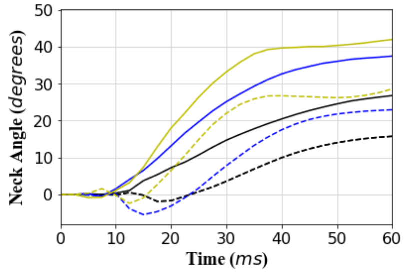

Koh (Fig. 2B)

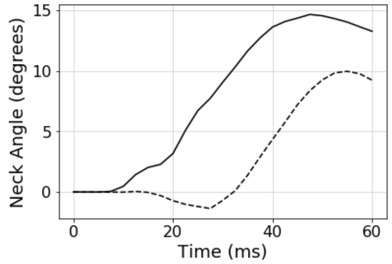

45

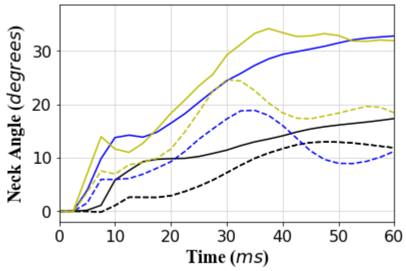

Kroell (Fig. 2D)

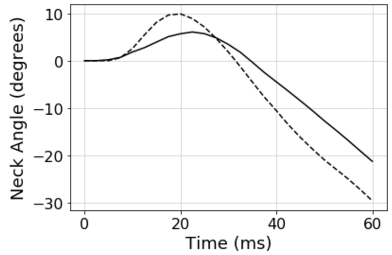

90

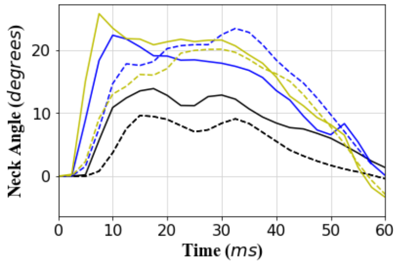

Hardy (Fig. 2C)

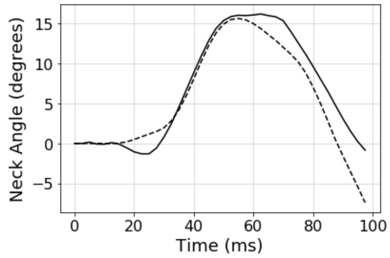

135

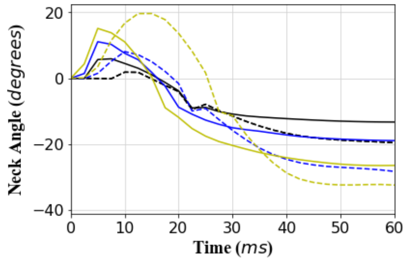

Viano (Fig. 2E)

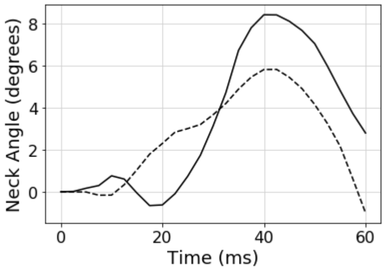

180

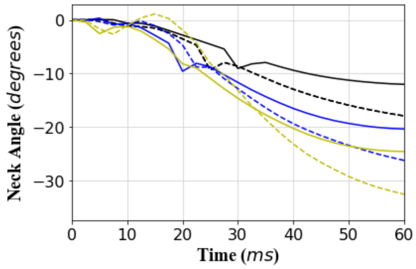

Forman (Fig. 2F)

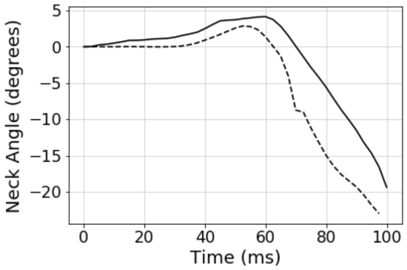

NBDL8 (Fig. 2G)

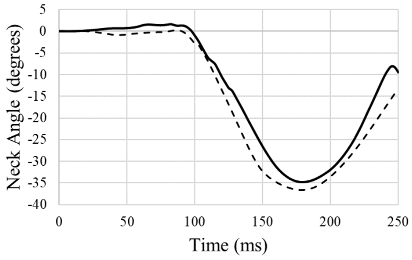

NBDL15 (Fig. 2G)

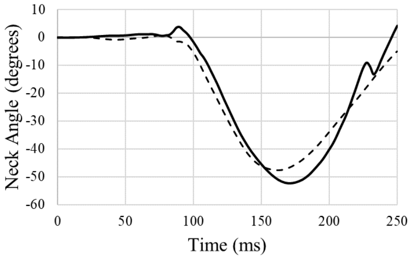

Figure A5: Neck Angle. M50-O (dashed), M50-OS (solid) at 3 (blk), 6 (blu), and 9 m/s (yel)
